# Tyrosinase-Mediated Oxidation of Endocannabinoid and Endovanilloid N-Arachidonoyl Dopamine and N-Arachidonoyl Tyrosine

**DOI:** 10.3390/biom16071040

**Published:** 2026-07-16

**Authors:** Alessia Mariano, Davide Laurenti, Antonio Francioso, Luciana Mosca, Anna Scotto d’Abusco, Mario Fontana

**Affiliations:** 1 Department of Biochemical Sciences, Sapienza University of Rome, 00185 Roma, Italy; alessia.mariano@uniroma1.it (A.M.); davide.laurenti@uniroma1.it (D.L.); luciana.mosca@uniroma1.it (L.M.); 2 Department of Bioscience and Technology for Food Agriculture and Environment, University of Teramo, 64100 Teramo, Italy; afrancioso@unite.it

**Keywords:** endocannabinoids, NADA, endovanilloids, tyrosinase, S-cysteinyl conjugates, pheomelanin, neurodegeneration

## Abstract

Endocannabinoids are lipid mediators consisting of esters, amides and ethers of long-chain polyunsaturated fatty acids. In this work, attention was focused on N-arachidonoyl tyrosine (NA-Tyr) and N-arachidonoyl dopamine (NADA), the amides of arachidonic acid with tyrosine and dopamine, respectively. NADA is an endogenous ligand of both type 1 cannabinoid receptors and type 1 vanilloid channel receptors. NADA is considered an endogenous compound with capsaicin-like activity and is distributed in several brain areas. The metabolic fate of endocannabinoids involves numerous enzymatic activities, which are only partially characterized. In particular, the biological activity of these biomolecules is terminated by enzymes with hydrolytic or oxygenase/oxidase activity. As part of this problem, we studied the oxidation of NADA and NA-Tyr mediated by mushroom tyrosinase. Our experimental data show that tyrosinase can oxidize both NADA and NA-Tyr. The oxidation of these biomolecules was also carried out in the presence of cysteine, allowing us to observe the formation of endocannabinoid/endovanilloid adducts with cysteine. These results were derived from chromatographic analyses and mass spectral experiments. During the tyrosinase-mediated oxidation in the presence of cysteine, it was possible to observe the production of a melanin-like pigment. The spectral characteristics of this pigment are consistent with those of pheomelanin, the pigment that contributes to the structure of neuromelanin. While mushroom tyrosinase serves here as a convenient biomimetic model to investigate the oxidative susceptibility of NADA and NA-Tyr, extrapolating these in vitro findings to mammalian physiology requires caution. Nevertheless, considering the neuronal distribution of these precursors and the documented, albeit debated, presence of tyrosinase-like activity in the central nervous system (CNS), these results offer a chemical rationale to further investigate whether similar oxidative pathways occur in vivo and potentially contribute to neurodegenerative mechanisms.

## 1. Introduction

Endocannabinoids are lipid mediators consisting of esters, amides and ethers of long-chain polyunsaturated fatty acids [1,2]. Among endogenous cannabinoids, N-arachidonoyl dopamine (NADA) and N-arachidonoyl tyrosine (NA-Tyr) are the amides of arachidonic acid with dopamine and tyrosine, respectively.

NADA is an endogenous ligand of both type 1 cannabinoid receptors (CB1) and the type 1 vanilloid channel receptor (TRPV1). NADA is considered as a member of the endovanilloid family with capsaicin-like activity and is distributed in several brain areas of the central nervous system such as the striatum, hippocampus and cerebellum [3,4]. Conversely, NA-Tyr is one of the possible precursors of the metabolic pathway leading to biosynthesis of NADA in vivo [5].

The formation and inactivation of NADA as well as its significance under physiological and pathological conditions are not fully understood yet. Nonetheless, there is evidence that NADA plays an important role in nociception and inflammation in the central and peripheral nervous system [6].

**Figure 1:**
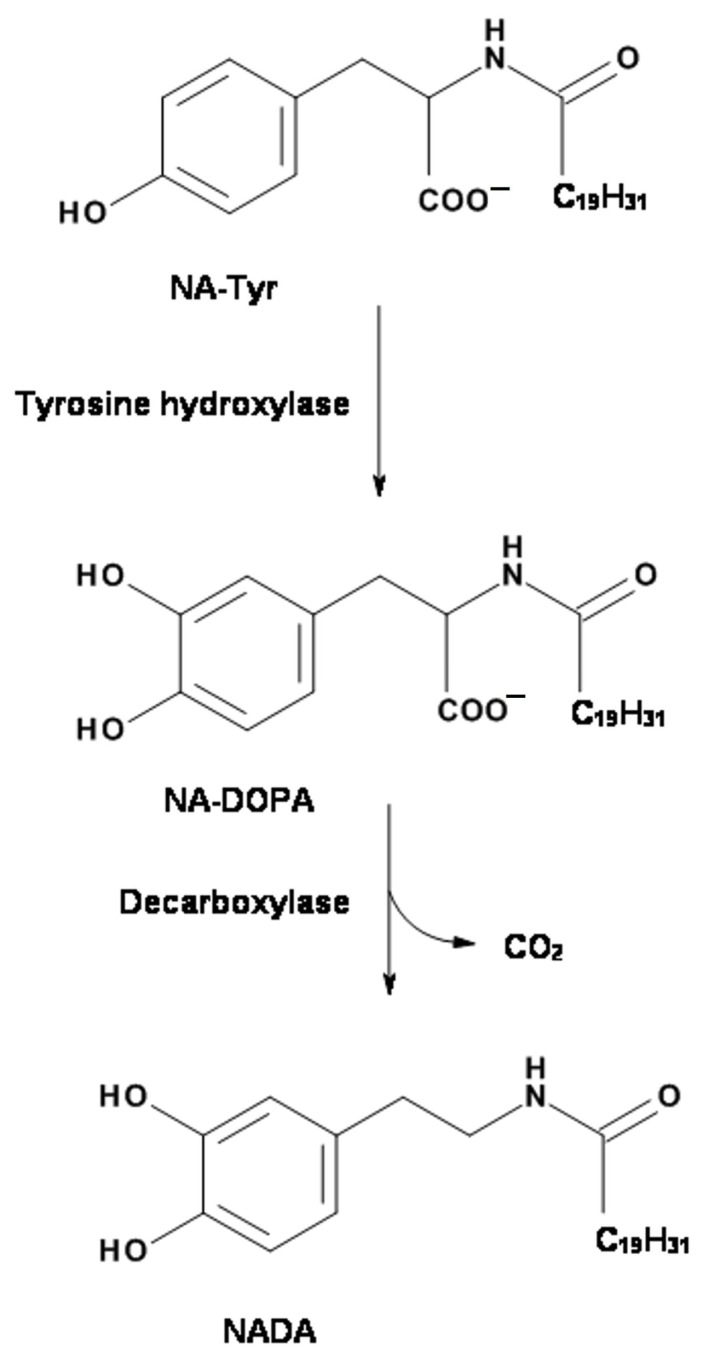
NADA biosynthesis pathway.

To date, numerous metabolic pathways have been described that lead to the biosynthesis of endocannabinoids [7,8,9]. NADA synthesis was observed almost exclusively in dopaminergic terminals. The first proposed pathway for NADA biosynthesis requires tyrosine hydroxylase and L-amino acid decarboxylase starting from NA-Tyr as a precursor and NA-DOPA as an intermediate (Figure 1) [5]. Similarly, the metabolic fate of endocannabinoids also involves numerous enzymatic activities, which are only partially characterized [10]. In particular, the biological activity of these biomolecules is terminated by enzymes with hydrolytic activity or alternatively by enzymes with oxygenase or oxidase activity [11]. To address this knowledge gap, we studied the enzymatic oxidation of NADA and NA-Tyr mediated by mushroom tyrosinase.

Tyrosinase is known to easily oxidize other substrates in addition to tyrosine, such as enkephalins or tetrahydroquinolines. A common feature in the structure of these molecules is the presence of a phenolic portion as enkephalins or a catecholic portion such as tetrahydroisoquinolines [12,13]. In particular, enkephalins are pentapeptides with opioid activity which possess tyrosine residue in the amino-terminal position. The action of tyrosinase on these bioactive peptides gives rise to the formation of melanin-like pigments which have been named opiomelanins [14,15]. These substrates are characterized by a phenolic or a catecholic moiety, similar to N-arachidonoyl derivatives of tyrosine and dopamine, respectively. Interestingly, opiomelanins are also generated by oxyradical attack on the tyrosine amino-terminal residue, which is converted to DOPA. The further oxidative modification of the DOPA residue produces opiomelanins, providing evidence for a possible route of melanin-like pigment synthesis without any enzyme intervention [16,17].

Tyrosinase-catalyzed oxidation of tyrosine generates the highly reactive dopaquinone [18]. Similarly, the oxidation of dopamine leads to the formation of quinones [19]. In the presence of cysteine, the quinones undergo nucleophilic addition with the concomitant production of adducts, mainly 5-S-cysteinyl DOPA and 5-S-cysteinyl dopamine. These adducts are successively converted through the intermediate synthesis of benzothiazine derivatives into pheomelanin pigments [20,21]. 5-S-cysteinyl dopamine, normally found in dopamine-rich regions of the brain, has been reported to act as an endogenous neurotoxin, contributing to the neurodegeneration of dopaminergic neurons in Parkinson’s disease [22]. Noteworthily, 5-S-cysteinyl dopamine units form the building blocks of neuromelanin, the characteristic pigment occurring in the bodies of dopaminergic neurons of substantia nigra [23]. Elemental analyses of neuromelanin extracted from substantia nigra indicate a high sulfur content and a molar C/H ratio indicating the presence of an aliphatic group and a benzothiazine moiety. The neuromelanin aliphatic component has been partially characterized as C_14_–C_20_ fatty acids. The complex composition of neuromelanin and the broad melanization encountered in these specific neuronal cells led to the hypothesis of a possible involvement of additional enzymes and substrates in the melanization process [24]. Along these lines, NADA and NA-Tyr upon oxidation can give rise to melanin-like pigments, contributing to neuromelanin production. Neuromelanin accumulates as a function of age in the normal human substantia nigra but is relatively depleted in the substantia nigra of patients with Parkinson’s disease [23]. The cytoprotective role of neuromelanin is controversial. Neuromelanin can also be pro-oxidant, becoming a source of free radicals. Moreover, neuromelanin can activate microglial cells, inducing the release of cytotoxic molecules that could damage other neurons and thus exacerbate the neurodegenerative process [25].

The aim of this work is to analyze the reaction products of tyrosinase-mediated oxidation of NA-Tyr and NADA and their reactivity in the presence of an excess of thiol as a strategy for the formation of new endocannabinoids. This research aims to shed light on the mechanisms underlying the formation of these biomolecules in order to understand their possible involvement in the complex inflammatory and nociceptive pathways underlying neurodegenerative diseases, but also to evaluate their potential as novel pharmaceutical targets in the fight against these diseases.

## 2. Materials and Methods

### 2.1. Materials

N-arachidonoyl tyrosine (NA-Tyr) and N-arachidonoyl dopamine (NADA) (Sigma Aldrich, St. Louis, MO, USA) were solubilized in 99.8% v/v ethanol at a concentration of 5 mM and 10 mM, respectively. Mushroom tyrosinase (1000 U/mL) (fungal tyrosinase, EC 1.14.18.1; catalogue no. T3824) and L-cysteine were provided by Sigma Aldrich. In our experimental conditions, a unit of mushroom tyrosinase was the amount of enzyme that increases the absorbance of tyrosine at 280 nm by 0.001 per minute at pH 6.5 at 25 °C. All other reagents and solvents were used with the highest level of commercial purity available.

### 2.2. NA-Tyr Oxidation: Chromatographic Analysis

Mushroom tyrosinase solution (50 U) was added to the reaction mixture containing 100 μM NA-Tyr and 50 mM phosphate buffer, pH 7.4 (1 mL final volume). The mixture was left in the dark at room temperature. Aliquots (150 μL) of the reaction mixture were chromatographically analyzed by HPLC at time zero and after 30, 60 and 120 min of incubation. A Waters chromatograph with a Waters 996 UV photodiode array detector (Waters corporation, Milford, MA, USA) was used for the analysis, setting the wavelength at 275 nm. The column was a Nova-pak C18 (3.9 mm × 150 mm), 4 μm (Waters corporation). The mobile phase consisted of two eluents: methanol/1 mM ammonium acetate (20:80 v/v), pH 6.8 (eluent A), and methanol/1 mM ammonium acetate (85:15 v/v), pH 6.8 (eluent B). The elution was carried out at a flow rate of 1 mL/min in a linear gradient from eluent A to 100% eluent B in 15 min. The peaks were identified using the calibration curve generated using known endocannabinoid concentrations.

### 2.3. NA-Tyr Oxidation: Spectrophotometric Analysis

For the oxidative reaction, reaction mixtures containing NA-Tyr from 5 to 100 μM, 100 U mushroom tyrosinase, and 50 mM phosphate buffer (pH 7.4) were prepared (1 mL final volume). The absorbance was measured at 400 nm over time to estimate the N-arachidonoyl dopaquinone formation. The spectrophotometric analysis was performed using a Perkin-Elmer Lambda 25 spectrophotometer (Perkin-Elmer, Shelton, CT, USA). The values of Km and Vmax of the reaction were calculated using GraphPad Prism 5.0 software (GraphPad Software, San Diego, CA, USA).

### 2.4. NADA Oxidation: Spectrophotometric Analysis

For the oxidative reaction, NADA from 10 to 250 μM, 100 U mushroom tyrosinase and 50 mM ammonium acetate buffer (pH 7.4) were mixed (1 mL final volume). The absorbance was measured at 400 nm over time to estimate the formation of N-arachidonoyl dopaminequinone species. The values of Km and Vmax of the reaction were calculated using the GraphPad Prism 5.0 software.

Spectral changes during NADA oxidation were recorded over time in the 230–800 nm range at room temperature. NADA was added to a mixture containing 20 U/mL mushroom tyrosinase and 50 mM ammonium acetate buffer, pH 6.8. Spectrophotometric analyses were performed using the Perkin-Elmer instrument (Lambda 25) (Perkin-Elmer).

### 2.5. N-Arachidonoyl 5-S-Cysteinyl DOPA Synthesis

A solution of NA-Tyr (100 μM) was incubated with 100 U of mushroom tyrosinase in 1 mL of 50 mM phosphate buffer (pH 7.4) in the presence of 200 μM L-cysteine. The absorbance was recorded at 6 min intervals in the range of wavelengths 230–400 nm using the Scan program of the Lambda 25 software associated with the Perkin-Elmer spectrophotometer (Perkin-Elmer).

### 2.6. N-Arachidonoyl 5-S-Cysteinyl Dopamine Synthesis

A solution of NADA (100 μM) was incubated with 100 U of mushroom tyrosinase in 1 mL of 50 mM ammonium acetate buffer (pH 6.8) in the presence of 200 μM L-cysteine. The absorbance was recorded at 6 min intervals in the range of 256–295 nm wavelengths using the Timedrive program of the Lambda 25 software associated with the Perkin-Elmer spectrophotometer (Perkin-Elmer).

### 2.7. NADA Oxidation: Chromatographic Analysis

To evaluate and quantify N-arachidonoyl 5-S-cysteinyl dopamine from NADA based on its mass-to-charge ratio (m/z), a chromatographic method was optimized using a Waters ACQUITY UPLC system (Waters corporation). The system included a quaternary solvent manager (QSM), a sample manager with a flow-through needle (FTN) system, a photodiode array detector (PDA), and a single-quadrupole mass detector with an electrospray ionization source (QDa).

The formation of N-arachidonoyl 5-S-cysteinyl dopamine product was evaluated after incubating NADA (100 μM) and 100 U of mushroom tyrosinase in 1 mL of 50 mM ammonium acetate buffer (pH 6.8) in the presence of 200 μM L-cysteine at room temperature for 40 min. After 40 min of reaction, the oxidation was stopped by acidifying the mixture to pH 1 with HCl.

Chromatographic analyses were performed to verify the m/z signal for N-arachidonoyl 5-S-cysteinyl dopamine, confirming the signal at an m/z of 557.82, in negative ionization mode. A NADA precursor was observed at m/z 440.6 in positive ionization mode with no detection of N-arachidonoyl 5-S-cysteinyl dopamine product.

For the chromatographic analysis, a Kinetex EVO C18column (1.7 µm, 100 Å, 2.1 × 100 mm, Phenomenex, Inc., Torrance, CA, USA) was used, thermostated at 25 °C, with a flow rate of 0.5 mL/min. Mobile phase A consisted of water with 0.1% formic acid, while mobile phase B was acetonitrile with 0.1% formic acid (Table 1).

### 2.8. Pheomelanin Synthesis from Endocannabinoids

Pheomelanin was synthesized from NA-Tyr or NADA. A solution containing 0.5 μmol of NA-Tyr or NADA was incubated with 200 U of mushroom tyrosinase in 1 mL of 50 mM phosphate buffer, pH 7.4. After 30 s, 1 μmol of L-cysteine was added. The reaction mixture was left for 24 h at room temperature, then acidified to pH 2.0 with 1 M HCl. The resulting solution was centrifuged for 30 min at 12,000 rpm to collect the pigment. The precipitated pigment was washed with 1 M HCl and subsequently dissolved in 1 mL of 1 M KOH. The identification of pheomelanin as a synthesis product was performed by analyzing the absorption spectrum (λ = 200–800 nm) of a solution containing 50 μL of the pigment previously dissolved in 1 M KOH in 50 mM phosphate buffer, pH 7.4. Spectral analysis was carried out using a Perkin-Elmer Lambda 25 UV-Vis spectrophotometer (Perkin-Elmer).

### 2.9. Statistical Analysis

The enzyme kinetic parameters Vmax and Km were determined by non-linear regression analysis fitting the initial velocity data to the Michaelis–Menten equation using GraphPad Prism 5.0 software. Data are expressed as mean ± standard error of the mean (SEM) from at least three independent experiments. Statistical comparisons between different substrates for kinetic parameters and product formation rates were performed using one-way analysis of variance (ANOVA). A *p*-value of less than 0.05 (*p* < 0.05) was considered statistically significant.

## 3. Results

### 3.1. NA-Tyr Oxidation: Kinetic Analysis

**Figure 2:**
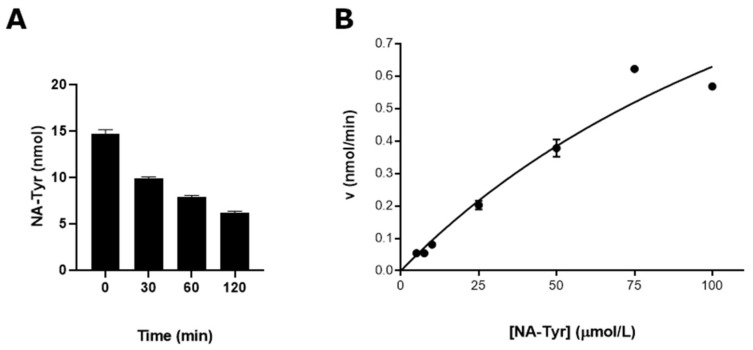
Tyrosinase-mediated NA-Tyr oxidation. (**A**) NA-Tyr concentration decreases as a function of time determined by HPLC analysis. (**B**) Curve for the NA-Tyr oxidation spectrophotometrically monitored at 400 nm. Individual data points represent experimental measurements, error bars indicate standard error of the mean (SEM), and the solid line represents the non-linear regression fit. Data are expressed as mean ± SEM of three independent experiments.

The oxidation of NA-Tyr mediated by mushroom tyrosinase was carried out under dark conditions at room temperature in 50 mM phosphate buffer, pH 7.4, and was monitored by chromatographic analysis. The HPLC analysis showed a decrease in the peak area with a retention time of 9 min, corresponding to the NA-Tyr. This decrease in peak area was time-dependent: it was observed that in 2 h approximately 67% of the endocannabinoid was oxidized (Figure 2A).

To study the tyrosinase-mediated oxidation of NA-Tyr, the reaction rate at 400 nm was monitored. This wavelength corresponds to the absorption of the dopaquinone species, which is formed following the catecholic activity of tyrosinase on the endocannabinoid NA-Tyr. Figure 2B shows the Michaelis–Menten curve for the NA-Tyr oxidation.

Under the same experimental conditions, the kinetic parameters of tyrosinase-mediated oxidation were evaluated for both NA-Tyr and L-Tyrosine (L-Tyr) as substrates. These parameters are reported in Table 2. Comparison of the obtained values of Km reveals that NA-Tyr shows a slightly higher affinity than L-Tyr for the enzyme, indicating similar substrate recognition. However, the NA-Tyr kcat value is markedly lower, resulting in an approximately 51-fold lower catalytic efficiency (kcat/Km) compared to L-Tyr. This demonstrates that, despite a comparable affinity and an efficient binding, NA-Tyr is converted much more slowly, making it a very poor substrate for tyrosinase catalysis.

### 3.2. NADA Oxidation: Kinetic Analysis

**Figure 3:**
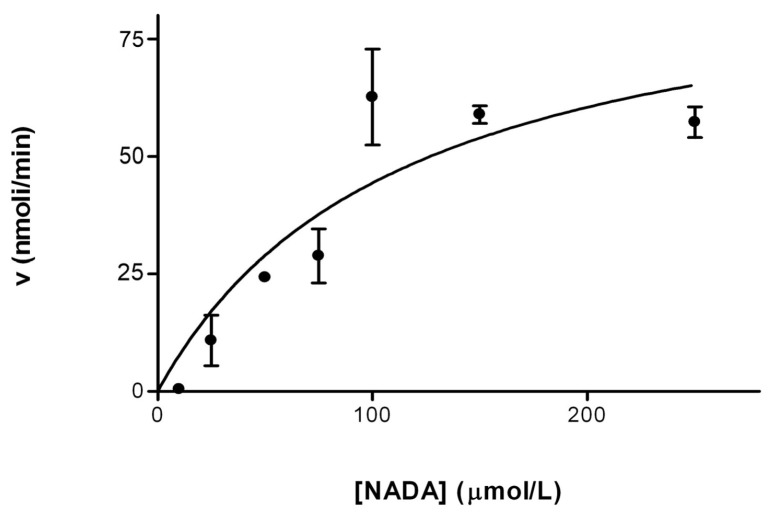
Tyrosinase-mediated NADA oxidation. Curve for the NADA oxidation spectrophotometrically monitored at 400 nm. Individual data points represent experimental measurements, error bars indicate standard error of the mean (SEM), and the solid line represents the non-linear regression fit. Data are expressed as mean ± SEM of three independent experiments.

Similarly to NA-Tyr, the tyrosinase-mediated enzymatic oxidation of the endocannabinoid/endovanilloid NADA was spectrophotometrically analyzed at 400 nm following the reaction rate. Figure 3 shows the Michaelis–Menten curve for NADA.

Under the same experimental conditions, the kinetics of oxidation of dopamine (DA) were evaluated.

The kinetic parameters of the reaction with NADA and with DA, obtained experimentally, are reported in Table 3. Comparison of the obtained values of Km reveals that NADA has about 4.5-fold lower affinity than dopamine for the enzyme, but retains a similar kcat value, leading to a moderate reduction in catalytic efficiency (~3.8-fold) compared to dopamine. These data suggest that the reduced affinity of NADA is partially compensated by an efficient catalytic turnover.

**Figure 4:**
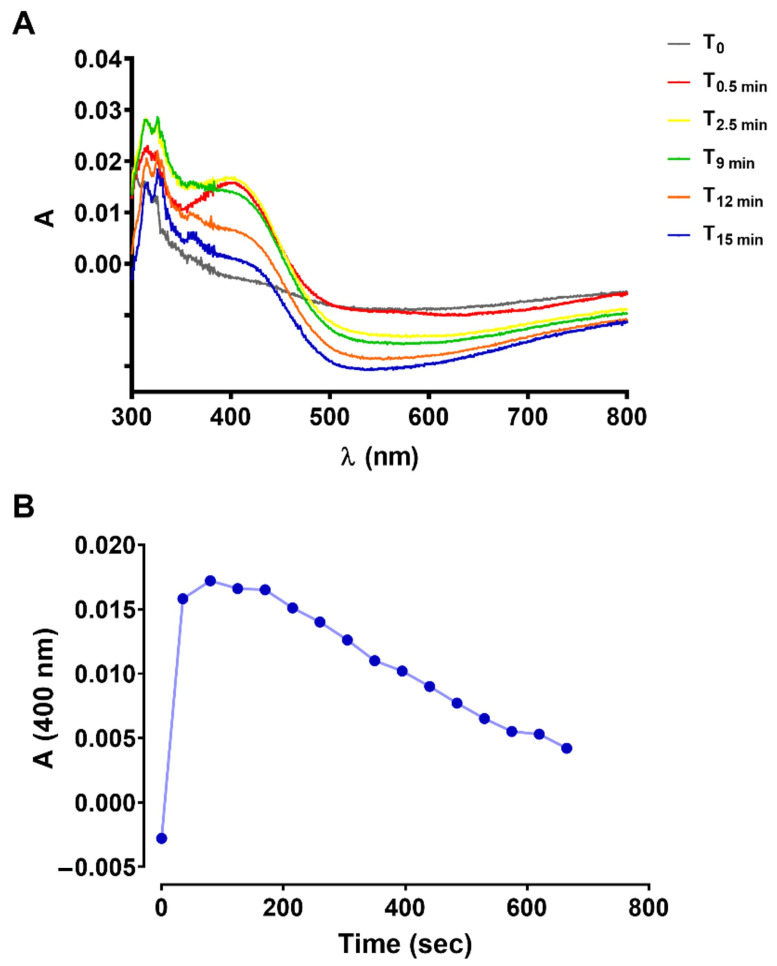
Tyrosinase-mediated NADA oxidation. (**A**) Spectral analysis of NADA in the range of 300–800 nm. (**B**) NADA absorbance over time at 400 nm. The absorbance (A) was monitored.

The tyrosinase-mediated NADA oxidation was followed spectrophotometrically over time in the wavelength range between 220 and 800 nm (Figure 4A). Spectral analysis revealed an alteration in the spectrum of NADA around 400 nm. Monitoring the NADA absorbance over time at 400 nm, an absorbance increase can be seen after about 2 min of analysis, followed by a slower decrease (Figure 4B).

### 3.3. N-Arachidonoyl 5-S-Cysteinyl DOPA and N-Arachidonoyl 5-S-Cysteinyl Dopamine Generation

**Figure 5:**
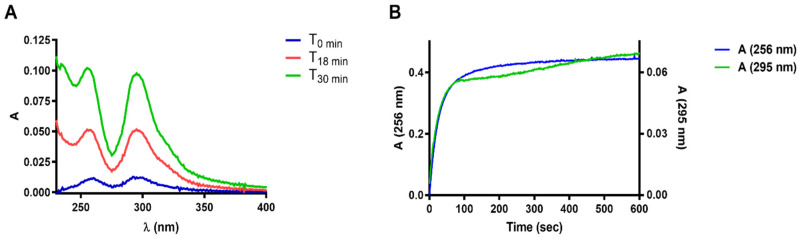
Tyrosinase-mediated NA-Tyr and NADA oxidation with cysteine. (**A**) Spectral analysis of N-arachidonoyl 5-S-cysteinyl DOPA generation by NA-Tyr oxidation in the range of 220–400 nm. (**B**) N-arachidonoyl 5-S-cysteinyl dopamine generation by NADA oxidation over time, spectrophotometrically monitored at 256–295 nm. The absorbance (A) was monitored.

It is known that the quinone species produced by the oxidative reaction of tyrosine catalyzed by mushroom tyrosinase can react with L-cysteine [26]. The product of this reaction is S-cysteinyl DOPA. Similarly, the incubation of NA-Tyr with tyrosinase in the presence of cysteine led to the formation of a thiol adduct. Figure 5A shows the spectral modifications of NA-Tyr during the oxidation reaction in the presence of cysteine. During the reaction, two absorption peaks appeared at the wavelengths of 256 nm and 295 nm. The formation of these peaks is indicative of the production of a NA-DOPA S-cysteinyl conjugate from NA-Tyr (Figure 5A).

Similarly, NADA was enzymatically oxidized in the presence of L-cysteine. To verify the formation of the thiol adduct, the absorbance was measured over time at 256 nm and 295 nm, the absorption wavelengths of N-arachidonoyl 5-S-cysteinyl dopamine (Figure 5B).

**Figure 6:**
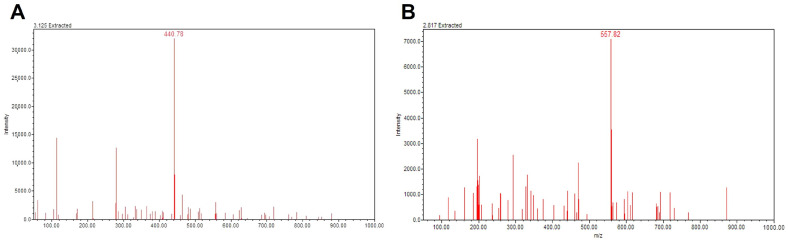
Tyrosinase-mediated NADA oxidation with cysteine. Chromatographic and mass spectral analysis of N-arachidonoyl 5-S-cysteinyl dopamine generation by NADA oxidation. (**A**) NADA spectrum and (**B**) N-arachidonoyl 5-S-cysteinyl dopamine spectrum.

N-arachidonoyl 5-S-cysteinyl dopamine generation by NADA oxidation was verified by performing a chromatographic analysis which allowed the observation of a product with a m/z signal at 557.82, which corresponded to N-arachidonoyl 5-S-cysteinyl dopamine formation from tyrosinase-mediated NADA oxidation in the presence of cysteine (Figure 6B). NADA was observed with a m/z signal at 440.6 (Figure 6A).

### 3.4. Pheomelanin Formation

**Figure 7:**
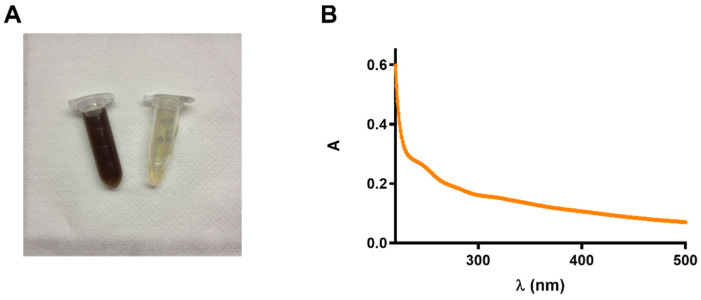
Pheomelanin formation. (**A**) Pigments from NA-Tyr and NADA. (**B**) Absorption spectrum pigment resulting from the NA-Tyr reaction in presence of cysteine. The absorbance (A) was monitored.

When the endocannabinoids, NA-Tyr and NADA, are incubated with L-cysteine in the presence of mushroom tyrosinase for 24 h an insoluble pigment is formed. This pigment appears brown for NA-Tyr and yellowish for NADA (Figure 7A).

The insoluble pigment derived from NA-Tyr was dissolved in 1 M KOH and subjected to UV-Vis spectrophotometric analysis. The absorption spectrum of this pigment resulting from the NA-Tyr reaction in the presence of cysteine (Figure 7B) showed an absorption maximum at about 230 nm. The spectral characteristics of this pigment are consistent with those of pheomelanin produced by tyrosine and dopamine and reported in the literature [27,28].

## 4. Discussion

The biosynthetic pathways leading to the formation of endocannabinoids in tissues have only been partially characterized to date. Alternative pathways concerning both the biosynthesis and metabolic fate of these biomolecules are currently being studied. The results reported in this work indicate that the endocannabinoids NA-Tyr and NADA undergo an oxidative metabolism catalyzed by the enzyme tyrosinase. Following the action of tyrosinase, the formation of oxidized derivatives of the tyrosine moiety in NA-Tyr and the dopamine moiety in NADA are formed. Moreover, the formation of a thioether-catechol derivative of NADA and NA-Tyr was observed in the presence of cysteine. In recent years, numerous endocannabinoids have been identified and synthesized, but cysteine derivatives of the endocannabinoids, which are produced by the nucleophilic addition of cysteine to the phenolic and catechol rings of NA-Tyr and NADA, respectively, have never been described until now. While the oxidation of free dopamine to cysteinyl-dopamine adducts is a well-established pathway in neurodegeneration, previous studies on acylated derivatives, such as NA-Tyr, or endocannabinoids, such as anandamide, have exclusively focused on either hydroperoxide/epoxide or generic polymer formation, completely missing the characterization of specific S-cysteinyl conjugates on the catechol head [22,29,30]. Our experimental data allow us to hypothesize a new oxidative metabolic fate for this type of endocannabinoid.

**Figure 8:**
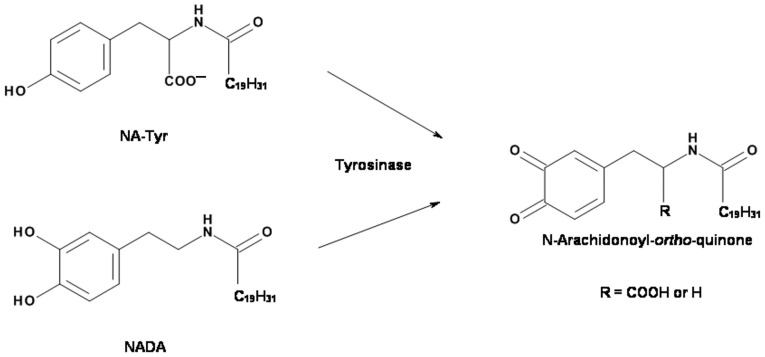
N-arachidonoyl-*ortho*-quinone formation by tyrosinase-mediated oxidation of NA-Tyr and NADA. Our results from Figure 4 confirmed the formation of N-arachidonoyl-*ortho*-quinone from NADA. The reaction product from NA-Tyr is only speculative.

We therefore tested the action of tyrosinase on these endocannabinoids, NA-Tyr and NADA. The kinetic parameters of tyrosinase-mediated oxidation of NA-Tyr and NADA do not differ substantially from those of tyrosine or dopamine, and it is therefore evident that both endocannabinoids can readily replace tyrosine and dopamine as substrates for tyrosinase (Figure 8). Although NA-Tyr and NADA are both accepted as substrates by tyrosinase, their kinetic behaviour differs significantly from that of L-tyrosine and dopamine. In particular, NA-Tyr shows a Km value comparable to that of L-Tyr, indicating similar substrate recognition and binding affinity. However, its kcat value is markedly lower, resulting in an approximately 51-fold lower catalytic efficiency (kcat/Km). This indicates that, despite efficient binding, NA-Tyr is converted much more slowly by the enzyme. By contrast, NADA displays lower apparent affinity than dopamine (higher Km), but retains a similar kcat value, leading to a more moderate reduction in catalytic efficiency (~3.8-fold). Therefore, our data support the conclusion that NA-Tyr and NADA can act as alternative substrates for tyrosinase, but not with the same catalytic efficiency as the native substrates.

Tyrosinase is the main and undoubtedly fundamental enzyme involved in the production of melanin. The formation of the melanin pigment requires the initial oxidation of phenolic and catechol precursors to *ortho*-quinones. Tyrosine is first hydroxylated by tyrosinase to DOPA, which is subsequently oxidized to dopaquinone. In the process of melanogenesis, the *ortho*-quinone thus formed undergoes a spontaneous intramolecular cyclization giving rise to the indole precursors of melanins [31].

**Figure 9:**
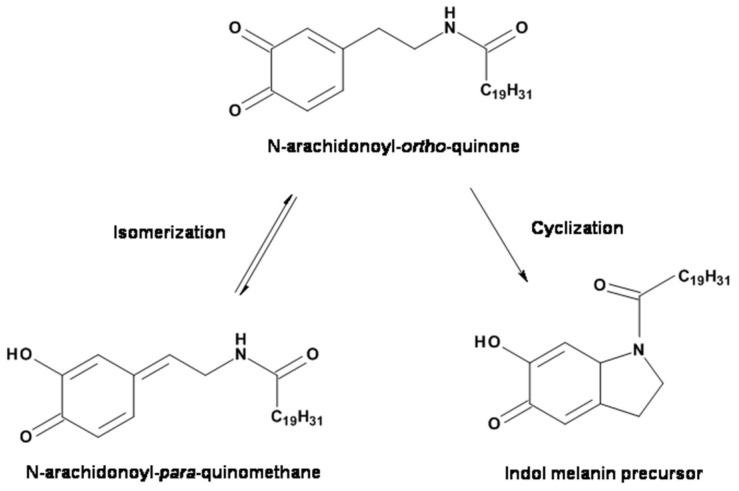
N-Arachidonoyl-ortho-quinone isomerization and cyclization reactions. Previous works demonstrated an analogue isomerization and cyclization process [31,33,34].

However, *ortho*-quinone can also isomerize to form *para*-quinomethane [32] (Figure 9). The isomerization pathway of *ortho*-quinone leads to the so-called sclerotization process that is observed in insects [33,34]. In insects, this process is triggered by the oxidation of the catechol precursor N-acetyldopamine which causes the hardening of the exoskeleton [35]. *Ortho*-quinone has a characteristic absorption spectrum with λmax at 400 nm. In our experimental conditions, the spectrophotometric analysis of the oxidation of NADA in the presence of tyrosinase revealed the rapid formation of an absorption maximum at 400 nm, indicating the formation of a compound with the spectral characteristics of *ortho*-quinone. This absorption decays slowly, suggesting that the *ortho*-quinone initially formed undergoes a subsequent transformation. As with other catechol precursors, the resulting N-arachidonoyl-*ortho*-quinone can cyclize or isomerize. It is possible that, due to the presence of the carbonyl group of the arachidonoyl tail linked to the nitrogen of dopamine, the isomerization of *ortho*-quinone is favoured over the cyclization. In NADA, the nitrogen atom is engaged in an amide linkage with the arachidonoyl tail. This conversion from a primary amine to an amide deeply decreases the lone pair availability on the nitrogen, rendering it non-nucleophilic under physiological/experimental pH and completely preventing spontaneous intramolecular cyclization. This proposed mechanism is supported by the experimental evidence that the prolonged oxidation of both NA-Tyr and NADA by tyrosinase does not lead to the formation of a detectable amount of melanin-like pigment.

**Figure 10:**
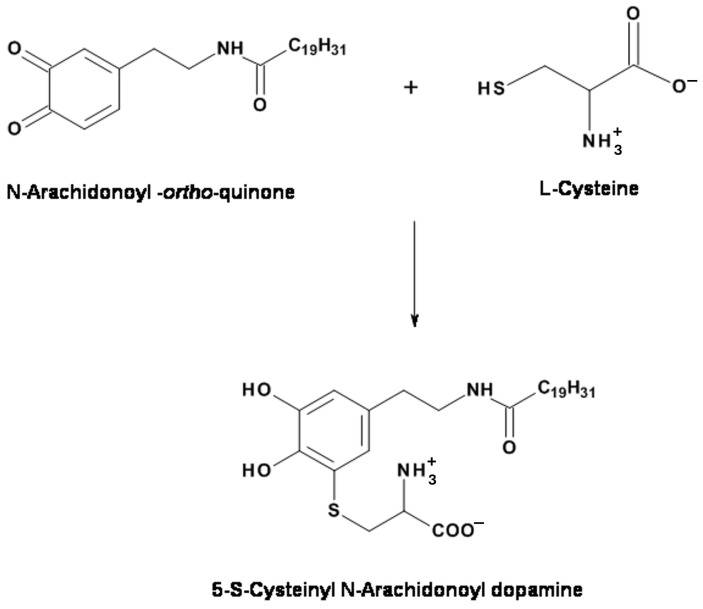
5-S-cysteinyl N-Arachidonoyl dopamine formation. Our results from Figure 5 and Figure 6 confirmed the formation of 5-S-cysteinyl N-arachidonoyl dopamine.

It is interesting to note that in the presence of thiol groups, the dopaquinone produced by the tyrosinase-mediated oxidation of tyrosine undergoes a nucleophilic addition with concomitant formation of adducts such as S-cysteinyl DOPA. These adducts are subsequently converted through the synthesis of an intermediate derived from benzothiazine into pigments of the pheomelanin type. Normally, tyrosinase is inhibited by sulfhydryl compounds that are capable of binding copper at the active site [36]. However, in the presence of an appropriate amount of thiols, the enzyme is not inhibited and the thiol reacts rapidly with the *ortho*-quinone formed, giving rise to adducts through the attack of the SH group on the aromatic ring (Figure 10). Herein, we observed that the oxidation of NA-Tyr with tyrosinase in the presence of cysteine leads to the formation of an adduct showing two UV absorption maxima at 256 and 295 nm, characteristic of the absorption of an S-cysteinyl DOPA moiety. Similarly, also the oxidation of NADA in the presence of cysteine with tyrosinase revealed a time-dependent increase in the absorption at 256 and 295 nm, characteristic of the formation of a thioether-catechol derivative of NADA. Mass spectral analysis confirmed the effective formation of the cysteinyl adduct with NADA. However, more detailed experiments are needed to better characterize the structure of the reaction product.

It is worth mentioning that when an *ortho*-benzoquinone intermediate undergoes a nucleophilic attack by a thiol (like L-cysteine) this preferentially yields the 5-S-conjugate as the major product, with minor amounts of the 2-S-conjugate [37]. The alkyl group at position 1 acts as a weak inductive electron-donating group, which slightly deactivates positions 2 and 6. Consequently, nucleophilic attack by the cysteine thiol group occurs preferentially at C-5 (and to a lesser degree at C-2), leading to 5-S-cysteinyldopa or 5-S-cysteinyldopamine as the major product.

As with other phenolic or catechol substrates, in addition to cysteine, other thiols such as glutathione can also react with the dopaquinone derivative of NA-Tyr and NADA. The nucleophilic addition reaction on *ortho*-quinone is very fast. For this reason, only the enzymatic oxidation of NA-Tyr or NADA to *ortho*-quinone is essential for thiol binding to the endocannabinoid. We have in fact observed that the rate of formation of the S-cysteinyl conjugate is the same as that of formation of the quinone which is obtained from the oxidation of NA-Tyr with tyrosinase in the absence of thiols.

Contrary to what occurs in the absence of cysteine, the action of tyrosinase on both NA-Tyr and NADA in the presence of cysteine induces the formation of a pigment with the spectral characteristics of pheomelanin. This result indicates that the adduct formed between the endocannabinoid and cysteine following the action of tyrosinase is in turn transformed into quinone, which undergoes intramolecular cyclization forming a benzothiazine core. The various benzothiazine units copolymerize with each other producing the pheomelanin-like pigment that we have observed. These data are encouraging in suggesting the formation of this pigment; however, EPR spectroscopy, or advanced spectroscopic methods, will unequivocally demonstrate the pheomelanin formation. It is interesting to note that neuromelanin, the pigment present in some brain cells (substantia nigra and locus coeruleus) shares many chemical and structural properties with pheomelanin. Neuromelanin is a mixed type of melanin in whose structure aliphatic groups, benzothiazines and indole are present [38,39,40]. It is possible, but remains to be demonstrated, that endocannabinoids as well as other neuromodulators already reported in the literature, such as enkephalins, contribute to the formation of neuromelanin [41].

The entry into cells of cysteine or glutathione constitutes the first step in the field of phenomena related to oxidative stress. For this reason, the in vivo formation of these thioether-catechol derivatives cannot be excluded, particularly in the brain where both the tyrosinase and the endocannabinoids/endovanilloids studied in this work are present. In this work, we used mushroom tyrosinase, instead of the human one, since it is a well-characterized and widely used oxidizing enzyme for studying the oxidation of phenolic and catecholic substrates, allowing controlled investigation of the chemical reactivity and product formation of the compounds analyzed in this study. Our primary intention was not to demonstrate that the same reactions necessarily occur in vivo in the mammalian CNS, but rather to evaluate whether these substrates are chemically susceptible to tyrosinase-mediated oxidation under biologically relevant conditions. In the future we are planning to perform analysis using human experimental models.

The formation of these S-cysteinyl conjugates can also be induced directly by reactive oxygen and nitrogen species such as the hydroxyl radical or peroxynitrite [42]. Noteworthy is the experimental evidence that thioether derivatives of dopamine (5-S-cysteinyldopamine) act as neurotoxins [43]. Speculatively, NADA, whose cerebral distribution reflects that of dopaminergic neurons, could undergo the same reactions as dopamine, leading to the formation of the S-cysteinyl conjugate N-arachidonoyl-5-S-cysteinyl dopamine.

## 5. Conclusions

Our results allow us to hypothesize the potential presence in the tissues of two new groups of compounds, the endocannabinoid S-cysteinyl conjugates of NA-Tyr and NADA and the melanin-like pigment produced by the oxidation of these conjugates. The study and further characterization of these new S-cysteinyl conjugates of the endocannabinoids NA-Tyr and NADA can fill a significant gap in the understanding of endocannabinoid chemical biology, thereby opening new perspectives in understanding not only the metabolic and functional significance of these biomolecules but also their possible involvement in the complex pathological mechanisms underlying neurodegenerative diseases. This represents a significant achievement in the pharmaceutical field, as the identification of alternative metabolic products can open new horizons for the formulation of novel targeted therapeutics.

## Figures and Tables

**Table 1:** Chromatographic elution gradient.

Minute	Solvent A (%)	Solvent B (%)
0.00	98	2
1.00	98	2
6.00	45	55
10.00	20	80
10.50	0	100
12.50	0	100
13.00	98	2
17.00	98	2

**Table 2:** Kinetic parameters of tyrosinase-mediated oxidation of NA-Tyr and L-Tyr.

	NA-Tyr	L-Tyr
**Km**	174.30 ± 8.70 μM	218.60 ± 10.90 μM
**Vmax**	6.4 × 10^−1^ ± 0.06 nmol/min **	40.0 ± 4.0 nmol/min
**kcat**	0.005 ± 0.0006 s^−1^ **	0.317 ± 0.032 s^−1^
**kcat/Km**	(2.87 ± 0.41) × 10^−5^ μM^−1^ s^−1^	(1.45 ± 0.21) × 10^−3^ μM^−1^ s^−1^

** *p* < 0.01 Vmax NA-Tyr vs. L-Tyr and kcat NA-Tyr vs. L-Tyr.

**Table 3:** Kinetic parameters of tyrosinase-mediated oxidation of NADA and DA.

	NADA	DA
**Km**	110.60 ± 11.10 μM *	24.35 ± 2.50 μM
**Vmax**	78.0 ± 7.75 nmol/min	63.5 ± 6.40 nmol/min
**kcat**	0.60 ± 0.05 s^−1^	0.50 ± 0.06 s^−1^
**kcat/Km**	(5.42 ± 0.71) × 10^−3^ μM^−1^ s^−1^ *	(20.50 ± 3.2) × 10^−3^ μM^−1^ s^−1^

* *p* < 0.05 Km and kcat/Km NADA vs. DA.

## Data Availability

Data is contained within the article and is available on request.

## References

[1] Basavarajappa B. (2007). Neuropharmacology of the Endocannabinoid Signaling System-Molecular Mechanisms, Biological Actions and Synaptic Plasticity. Curr. Neuropharmacol..

[2] Afshar S., Abbasinazari M., Amin G., Farrokhian A., Sistanizad M., Afshar F., Khalili S. (2022). Endocannabinoids and Related Compounds as Modulators of Angiogenesis: Concepts and Clinical Significance. Cell Biochem. Funct..

[3] Grabiec U., Dehghani F. (2017). N -Arachidonoyl Dopamine: A Novel Endocannabinoid and Endovanilloid with Widespread Physiological and Pharmacological Activities. Cannabis Cannabinoid Res..

[4] Novosadova E., Dolotov O., Inozemtseva L., Novosadova L., Antonov S., Shimchenko D., Bezuglov V., Vetchinova A., Tarantul V., Grivennikov I. (2022). Influence of N-Arachidonoyl Dopamine and N-Docosahexaenoyl Dopamine on the Expression of Neurotrophic Factors in Neuronal Differentiated Cultures of Human Induced Pluripotent Stem Cells under Conditions of Oxidative Stress. Antioxidants.

[5] Hu S.S.-J., Bradshaw H.B., Benton V.M., Chen J.S.-C., Huang S.M., Minassi A., Bisogno T., Masuda K., Tan B., Roskoski R. (2009). The Biosynthesis of N-Arachidonoyl Dopamine (NADA), a Putative Endocannabinoid and Endovanilloid, via Conjugation of Arachidonic Acid with Dopamine. Prostaglandins Leukot. Essent. Fat. Acids.

[6] Huang S.M., Walker J.M. (2006). Enhancement of Spontaneous and Heat-Evoked Activity in Spinal Nociceptive Neurons by the Endovanilloid/Endocannabinoid N -Arachidonoyldopamine (NADA). J. Neurophysiol..

[7] van der Stelt M., Hansen H.H., Veldhuis W.B., Bär P.R., Nicolay K., Veldink G.A., Vliegenthart J.F.G., Hansen H.S. (2003). Biosynthesis of Endocannabinoids and Their Modes of Action in Neurodegenerative Diseases. Neurotox. Res..

[8] Simard M., Archambault A.-S., Lavoie J.-P.C., Dumais É., Di Marzo V., Flamand N. (2022). Biosynthesis and Metabolism of Endocannabinoids and Their Congeners from the Monoacylglycerol and N-Acyl-Ethanolamine Families. Biochem. Pharmacol..

[9] Muccioli G.G. (2010). Endocannabinoid Biosynthesis and Inactivation, from Simple to Complex. Drug Discov. Today.

[10] Bhandari S., Bisht K.S., Merkler D.J. (2022). The Biosynthesis and Metabolism of the N-Acylated Aromatic Amino Acids: N-Acylphenylalanine, N-Acyltyrosine, N-Acyltryptophan, and N-Acylhistidine. Front. Mol. Biosci..

[11] Giuffrida A., Beltramo M., Piomelli D. (2001). Mechanisms of Endocannabinoid Inactivation: Biochemistry and Pharmacology. J. Pharmacol. Exp. Ther..

[12] Rosei M.A., Antonilli L., Coccia R., Foppoli C. (1989). Enkephalins and Exorphins Oxidation by Tyrosinase. Biochem. Int..

[13] Ito S., Sugumaran M., Wakamatsu K. (2020). Chemical Reactivities of Ortho-Quinones Produced in Living Organisms: Fate of Quinonoid Products Formed by Tyrosinase and Phenoloxidase Action on Phenols and Catechols. Int. J. Mol. Sci..

[14] Rosei M.A. (1996). Melanins From Opioid Peptides. Pigment. Cell Res..

[15] Rosei M.A. (2001). Opiomelanins Synthesis and Properties. Histol. Histopathol..

[16] Fontana M., Mosca L., Rosei M.A. (2001). Interaction of Enkephalins with Oxyradicals11Abbreviations: ABAP, 2,2′-Azobis(2-Amidinopropane); Dopa, Dihydroxyphenyl-Alanine; H_2_O_2_, Hydrogen Peroxide; Leu-Enk, Leu-Enkephalin; Met-Enk, Met-Enkephalin; LOOH, Linoleic Acid 13-Hydroperoxide; NBT, Nitro Blu. Biochem. Pharmacol..

[17] Mosca L., De Marco C., Fontana M., Rosei M.A. (1999). Fluorescence Properties of Melanins from Opioid Peptides. Arch. Biochem. Biophys..

[18] Mine M., Mizuguchi H., Takayanagi T. (2022). Kinetic Analyses of Two-Steps Oxidation from l-Tyrosine to l-Dopaquinone with Tyrosinase by Capillary Electrophoresis/Dynamic Frontal Analysis. Anal. Biochem..

[19] Chakrabarti S., Bisaglia M. (2023). Oxidative Stress and Neuroinflammation in Parkinson’s Disease: The Role of Dopamine Oxidation Products. Antioxidants.

[20] Benathan M., Labidi F. (1996). Cysteine-Dependent 5-S-Cysteinyldopa Formation and Its Regulation by Glutathione in Normal Epidermal Melanocytes. Arch. Dermatol. Res..

[21] Mosca L., Lendaro E., D’Erme M., Marcellini S., Moretti S., Rosei M.A. (2006). 5-S-Cysteinyl-Dopamine Effect on the Human Dopaminergic Neuroblastoma Cell Line SH-SY5Y. Neurochem. Int..

[22] Badillo-Ramírez I., Saniger J.M., Rivas-Arancibia S. (2019). 5-S-Cysteinyl-Dopamine, a Neurotoxic Endogenous Metabolite of Dopamine: Implications for Parkinson’s Disease. Neurochem. Int..

[23] Zucca F.A., Giaveri G., Gallorini M., Albertini A., Toscani M., Pezzoli G., Lucius R., Wilms H., Sulzer D., Ito S. (2004). The Neuromelanin of Human Substantia Nigra: Physiological and Pathogenic Aspects. Pigment. Cell Res..

[24] Zecca L., Tampellini D., Gerlach M., Riederer P., Fariello R.G., Sulzer D. (2001). Substantia Nigra Neuromelanin: Structure, Synthesis, and Molecular Behaviour. J. Clin. Pathol.-Mol. Pathol..

[25] Moreno-García A., Kun A., Calero M., Calero O. (2021). The Neuromelanin Paradox and Its Dual Role in Oxidative Stress and Neurodegeneration. Antioxidants.

[26] Manini P., Napolitano A., Westerhof W., Riley P.A., D’Ischia M. (2009). A Reactive Ortho -Quinone Generated by Tyrosinase-Catalyzed Oxidation of the Skin Depigmenting Agent Monobenzone: Self-Coupling and Thiol-Conjugation Reactions and Possible Implications for Melanocyte Toxicity. Chem. Res. Toxicol..

[27] Pilas B., Sarna T., Kalyanaraman B., Swartz H.M. (1988). The Effect of Melanin on Iron Associated Decomposition of Hydrogen Peroxide. Free Radic. Biol. Med..

[28] Mariano A., Bigioni I., Scotto d’Abusco A., Baseggio Conrado A., Maina S., Francioso A., Mosca L., Fontana M. (2021). Pheomelanin Effect on UVB Radiation-Induced Oxidation/Nitration of l-Tyrosine. Int. J. Mol. Sci..

[29] Woodward D.F., Liang Y., Krauss A.H. (2008). Prostamides (Prostaglandin-ethanolamides) and Their Pharmacology. Br. J. Pharmacol..

[30] Burstein S.H., Rossetti R.G., Yagen B., Zurier R.B. (2000). Oxidative Metabolism of Anandamide. Prostaglandins Other Lipid Mediat..

[31] Ito S., Wakamatsu K. (2008). Chemistry of Mixed Melanogenesis—Pivotal Roles of Dopaquinone. Photochem. Photobiol..

[32] Qu Y., Zhan Q., Du S., Ding Y., Fang B., Du W., Wu Q., Yu H., Li L., Huang W. (2020). Catalysis-Based Specific Detection and Inhibition of Tyrosinase and Their Application. J. Pharm. Anal..

[33] Sugumaran M. (1998). Unified Mechanism for Sclerotization of Insect Cuticle. Adv. Insect Physiol..

[34] Kramer K.J., Kanost M.R., Hopkins T.L., Jiang H., Zhu Y.C., Xu R., Kerwin J., Turecek F. (2001). Oxidative Conjugation of Catechols with Proteins in Insect Skeletal Systems. Tetrahedron.

[35] Abebe A., Zheng D., Evans J., Sugumaran M. (2010). Reexamination of the Mechanisms of Oxidative Transformation of the Insect Cuticular Sclerotizing Precursor, 1,2-Dehydro-N-Acetyldopamine. Insect Biochem. Mol. Biol..

[36] Yap P.-G., Gan C.-Y. (2021). Multifunctional Tyrosinase Inhibitor Peptides with Copper Chelating, UV-Absorption and Antioxidant Activities: Kinetic and Docking Studies. Foods.

[37] Shen X.-M., Dryhurst G. (1996). Further Insights into the Influence of L-Cysteine on the Oxidation Chemistry of Dopamine: Reaction Pathways of Potential Relevance to Parkinson’s Disease. Chem. Res. Toxicol..

[38] Odh G., Carstam R., Paulson J., Wittbjer A., Rosengren E., Rorsman H. (1994). Neuromelanin of the Human Substantia Nigra: A Mixed-Type Melanin. J. Neurochem..

[39] Cai W., Wakamatsu K., Zucca F.A., Wang Q., Yang K., Mohamadzadehonarvar N., Srivastava P., Tanaka H., Holly G., Casella L. (2023). DOPA Pheomelanin Is Increased in Nigral Neuromelanin of Parkinson’s Disease. Prog. Neurobiol..

[40] Jakaria M., Cannon J.R. (2025). Neuromelanin-Induced Cellular Stress and Neurotoxicity in the Pathogenesis of Parkinson’s Disease. Apoptosis.

[41] Nagatsu T., Nakashima A., Watanabe H., Ito S., Wakamatsu K. (2022). Neuromelanin in Parkinson’s Disease: Tyrosine Hydroxylase and Tyrosinase. Int. J. Mol. Sci..

[42] Vauzour D., Ravaioli G., Vafeiadou K., Rodriguez-Mateos A., Angeloni C., Spencer J.P.E. (2008). Peroxynitrite Induced Formation of the Neurotoxins 5-S-Cysteinyl-Dopamine and DHBT-1: Implications for Parkinson’s Disease and Protection by Polyphenols. Arch. Biochem. Biophys..

[43] Aureli C., Cassano T., Masci A., Francioso A., Martire S., Cocciolo A., Chichiarelli S., Romano A., Gaetani S., Mancini P. (2014). 5-S-cysteinyldopamine Neurotoxicity: Influence on the Expression of A-synuclein and ERp57 in Cellular and Animal Models of Parkinson’s Disease. J. Neurosci. Res..

